# Chronic disease medication management at home: a quantitative survey among 180 patients

**DOI:** 10.3399/BJGPO.2024.0027

**Published:** 2024-11-13

**Authors:** Sabine Bayen, Yolaine Haegeman, Nassir Messaadi, Marc Bayen, Maurice Ponchant, Anthony Haro, François Quersin, Matthieu Calafiore

**Affiliations:** 1 Department of General Medicine, Université Lille, Faculty of Medicine, Lille, France; 2 Université Lille, CHU Lille, ULR 2694 - MSPU Guesnain, Lille, France; 3 Université Lille, CHU Lille, ULR 2694 - PSPU Lille, Lille, France; 4 Université Lille, CHU Lille, ULR 2694 - MSPU Wattrelos, Lille, France

**Keywords:** medication, chronic disease, management

## Abstract

**Background:**

In France, 40% of people aged >16 years (20 million) report having at least one chronic disease requiring long-term treatment. Compliance with treatment at home is estimated to be 50% on average.

**Aim:**

To study the practical management of oral treatments at home by people living with one or more chronic diseases.

**Design & setting:**

A quantitative, descriptive, observational, cross-sectional study. Thirty GPs in France were invited by email to enrol 10 consecutive patients with chronic diseases.

**Method:**

Standardised questionnaires were used to assess the sociodemographic profile of doctors and patients, and the management of oral medication at home.

**Results:**

Twenty GPs collected 180 questionnaires of which 70% responders said they did not find taking their medication a problem; 43% used a pillbox; 79% said they knew 'all' their medications; and 61% reported forgetting to take their medication (versus 30% who reported never forgetting to take their medication).

**Conclusion:**

More than half of patients are non-adherent to taking oral medication at home for their long-term conditions. Personalised reminders could reduce unintentional medication non-adherence.

## How this fits in

Research has shown compliance among people with chronic diseases to long-term treatment averages only 50% in general. This study asked 180 people with chronic illness how they manage their medication at home. Only 39% of responders never forgot to take their medication. The frequency of omissions remained the same with or without a pillbox. Research in primary care settings and focusing on concrete patient habits at home regarding medication management (MM) is still sparse but needed. It gives the GP insight to threats and opportunities of MM at home, and therefore could optimise medication adherence in chronic disease (CD) .

## Introduction

Polypathologies are prevalent and can be challenging for GPs owing to the increasing range of disorders diagnosed and treated as disease, and a lower threshold of range and severity of symptoms required for diagnosis and treatment. At the same time, GPs face patients’ changing perceptions of disease and expectations of treatment. Furthermore, polypathologies are becoming more common as the population ages.^1^ In 1960, 4.3% of the population in France was aged ≥75 years, which increased to 7.2% in 2000 and 9.6% in 2020. In 2019, 91% of people aged ≥75 years had at least one CD or treatment.^2^ In 2019, 37% of people aged ≥75 years had cardiovascular disease: 14% had coronary heart disease, 9% had heart failure, 7% had a stroke, and 16% had a rhythm disorder. Men were particularly affected (46% of people aged ≥75 years for all cardio-neurovascular pathologies, compared with 31% of women of the same age).^3^

In addition, the incidence of respiratory diseases is also increasing (12% of people aged ≥75 years in 2019).^4^

In France, 15% of women and 12% of men aged 16–19 years, almost half of those aged 55–59 years, and more than 70% of women and men aged ≥75 years report having a CD or health problem.^5^ People living with one or more CD are usually on long-term oral treatment to stabilise the course of their disease and maintain their quality of life. Therapeutic compliance is defined as the congruence between patient behaviour (for example, taking medication) and the prescribed treatment in the context of CD.^6,7^ According to the World Health Organization (WHO), compliance among patients with CD averages only 50% in high-income countries.^8^

GPs are at the forefront of supporting patients with CD, encouraging them to adhere to their treatment regimens daily, and preventing and identifying the risks associated with home MM.^9,10^

The main objective of this study was to find out about patients’ habits in taking their long-term medication, including the practical arrangements for doing so (storage, preparation, reminders).

## Method

### Study design

We conducted a quantitative, descriptive, observational, cross-sectional study. Standardised anonymised questionnaires were used to assess the sociodemographic profiles of physicians and patients, and the patients’ management of oral medication at home.

### Study population

The inclusion criteria for GPs were to work in private practice in the Nord Pas-de-Calais region, and the exclusion criteria were to be a hospital doctor or a locum.

The inclusion criteria for patients were to be adult (aged ≥18 years), to consent to participate in the study, and to have one or more CD undergoing long-term treatment. People with one or more CD without long-term treatment and people who refused to participate in the study were excluded.

### Recruitment

Convenience sampling was used, which aimed to include 300 patients, who were considered to be representative of this French region. Thirty GPs were invited by email to take part in the study. The email contained the questionnaires for both GPs and patients. GPs were asked to recruit 10 consecutive patients from their practices who met the inclusion criteria. After obtaining oral consent from the patients, the GP handed them the anonymised questionnaire to complete on their own or with the help of their GP. The questionnaire was also made available in the waiting room, so that patients could choose to complete it alone before the consultation, or with the GP during the consultation, so that they could complete all or part of the questionnaire together.

### Data collection

The questionnaires were distributed between 24 July 2023 and 3 October 2023. They were either handed to the GP, left in their letterbox, sent to their secretary after acceptance by telephone contact, or emailed the questionnaire to print. Before enrolling patients with chronic conditions, GPs also completed a questionnaire on their investigator profile. (sex, age, year of establishment, practice structure, urban or rural location, CD or not, use of pillbox). The questionnaires have been stored in a closed urn in the GP's office and have been collected by the second author.

### Questionnaire

The questionnaire was inspired by results from a previous qualitative study, which had a sample of 30 patients with Parkinson’s disease and other CDs.

The questionnaire was validated by the university's data protection officer and was tested by 10 patients. The questionnaire (Supplementary Information) consisted of the following five parts: (1) the chronic disease(s); (2) the pharmacy; (3) preparation of medicines at home; (4) compliance; (5) sociodemographic data. The questionnaire consisted of 35 standardised closed questions, except for 15, 27, and 33, which were open-ended.

### Data analysis

The data of this non-web-based survey have been transcribed in an Excel table. The χ^2^ test was used to test for dependence between variables. The significance level was set at 5% (*P*≤0.05). The data were calculated using XLSTAT in Excel.

## Results

### Characteristics of the GP population

Twenty out of 30 invited GPs participated in the study and practised in nine different areas of the Nord-Pas de Calais region in France.

Table 1 shows the sociodemographic characteristics of the GP population.

**Table 1.** Characteristics of the participating GPs

	Sex	Age	Installation	Type of practice	Area of practice	Chronic disease personally affected	Pillbox use
M1	F	49	2019	MDS	Urban	No	
M2	F	39	2019	MDS	Urban	No	
M3	M	43	2009	MDS	Rural	Yes	No
M4	M	35	2019	MDS	Urban	No	
M5	F	32	2019	MDS	Rural	No	
M6	M	60	1992	MDS	Rural	No	
M7	M	49	2006	MDS	Rural	No	
M8	F	47	2011	MDS	Urban	No	
M9	F	38	2016	MDS	Urban	No	
M10	F	37	2018	MDS	Rural	No	
M11	M	61	1992	CDG	Urban	Yes	Yes
M12	F	53	1998	MDS	Rural	No	
M13	M	32	2020	CDG	Rural	Yes	No
M14	F	32	2021	MDS	Urban	No	
M15	M	47	2005	CDG	Urban	No	
M16	M	31	2020	CDG	Urban	No	
M17	M	46	2007	CDG	Urban	No	
M18	F	42	2012	CDG	Urban	No	
M19	F	42	2010	MDS	Rural	Yes	No
M20	F	36	2021	CDG	Rural	No	

CDG = group practice. MSD = health centre

### Characteristics of the population with chronic illness

Table 2 shows the sociodemographic characteristics of patients.

**Table 2.** Characteristics of the participating people with chronic diseases

*n* = 180	*n*	%
**Sex**		
Male	86	48
Female	94	52
**Age**	66.5 years (18;93)	
>65 years	114	63
**Lifestyle**		
As a couple or with a family member	133	74
Professional activity	155	
Retired	121	67
Employee	21	11
No activity	13	7
Activity before retirement	78	
Employee	23	29
Worker	18	23
Managers and professionals	10	13
No activity	11	14
**Declared chronic disease**		
Hypertension	104	58
Type 2 diabetes	45	25
Dyslipidaemia	45	25
Rhythm disorders	41	23
Heart failure	32	18
Depression	27	15
Autoimmune diseases	27	15
Myocardial infarction	18	10
Cancer	18	10
Asthma	16	9
Cerebrovascular accident	16	9

### Age distribution among the population with chronic illness

**Figure 1.**
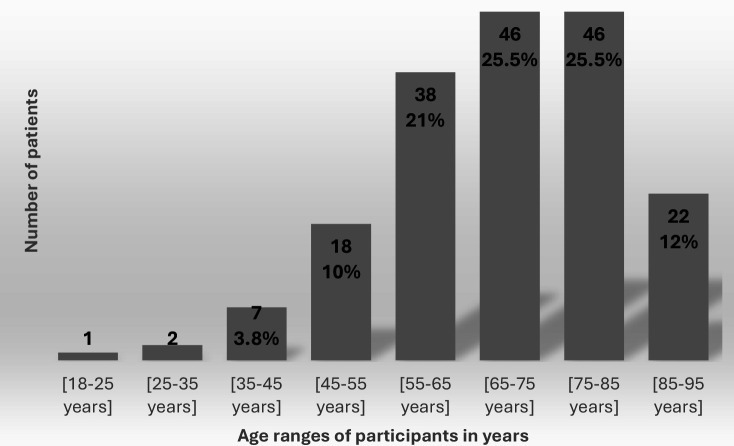
Age range of study participants

Figure 1 shows the age distribution of our study sample.

### Medication management and adherence

Table 3 shows how daily treatments are managed at home and reasons for omissions. For 70% of responders, taking daily treatment was not a burden. Experiencing treatment as a burden was independent of sex (*P* = 0.815 see Supplementary file).

**Table 3.** Terms of everyday medication management at home

*n* = **180**	*n*	%
**Who collects the medicines from the pharmacy?**		
The patients themselves	139	77
The spouse	35	19
A family member other than the spouse	15	8
Home delivery by pharmacy	4	2
The nurse	3	1.6
**Storage of medicines**	***n***	**%**
Medicine box	85	47
Plastic box	45	25
Medicine bag (paper or plastic)	20	11
Other: trays, worktop, handbag, drawer	14	8
**Drug storage facilities**	***n***	**%**
Kitchen	78	43
Bathroom	43	24
Living room	25	14
Bedroom	24	13
Cellar	10	5.5
Multiple locations	9	5
**Who prepares the medicines?**	***n***	**%**
The patients themselves	153	85
Spouse	13	7
Nurse	9	5
Family and friends	5	3
**Adherence to treatment**	***n***	**%**
Never forgot to take	70	39
Rarely forgot to take	68	38
Sometimes forgot to take	36	21
Often forgot to take	4	2
**Treatment reminder mode**	***n***	**%**
Out of habit	147	97
Home nurse	5	3
Telephone alarm	4	2
Alarm clock	3	1.6
At mealtime	3	1.6
Telephone application	1	0.5
**Frequency of forgetfulness with versus without a pillbox**	**With versus without**	**%**
Never	27 versus 43	35 versus 42
Rarely	31 versus 37	41 versus 36
Sometimes	16 versus 21	21 versus 20
Often	2 versus 1	3 versus 1
**Reported reasons for forgetting medication**	**Unintentional**	**Intentional**
*'I don't like taking pills.'*		1
*'I'm afraid of the side effects.'*		1
*'I feel good so I forget.'*	1	
*'Recent change in treatment.'*	1	
*'Memory problems; forgetting to prepare.'*	2	
*'Fatigue.'*	9	
*'Away from home, restaurant; travel.'*	25	
*'Concerns.'*	20	
*'Pace of life (coming home late from work).'*	19	
*'Distractions; pillbox out of sight.'*	13	
*'Aerosols'*		

Several answers were possible

Regarding the existence of possible conflicts or disputes related to treatment management, 153 (85%) said they never had them, 14 (8%) rarely, 12 (7%) sometimes, and the youngest participant, aged 18, often.

**Figure 2.**
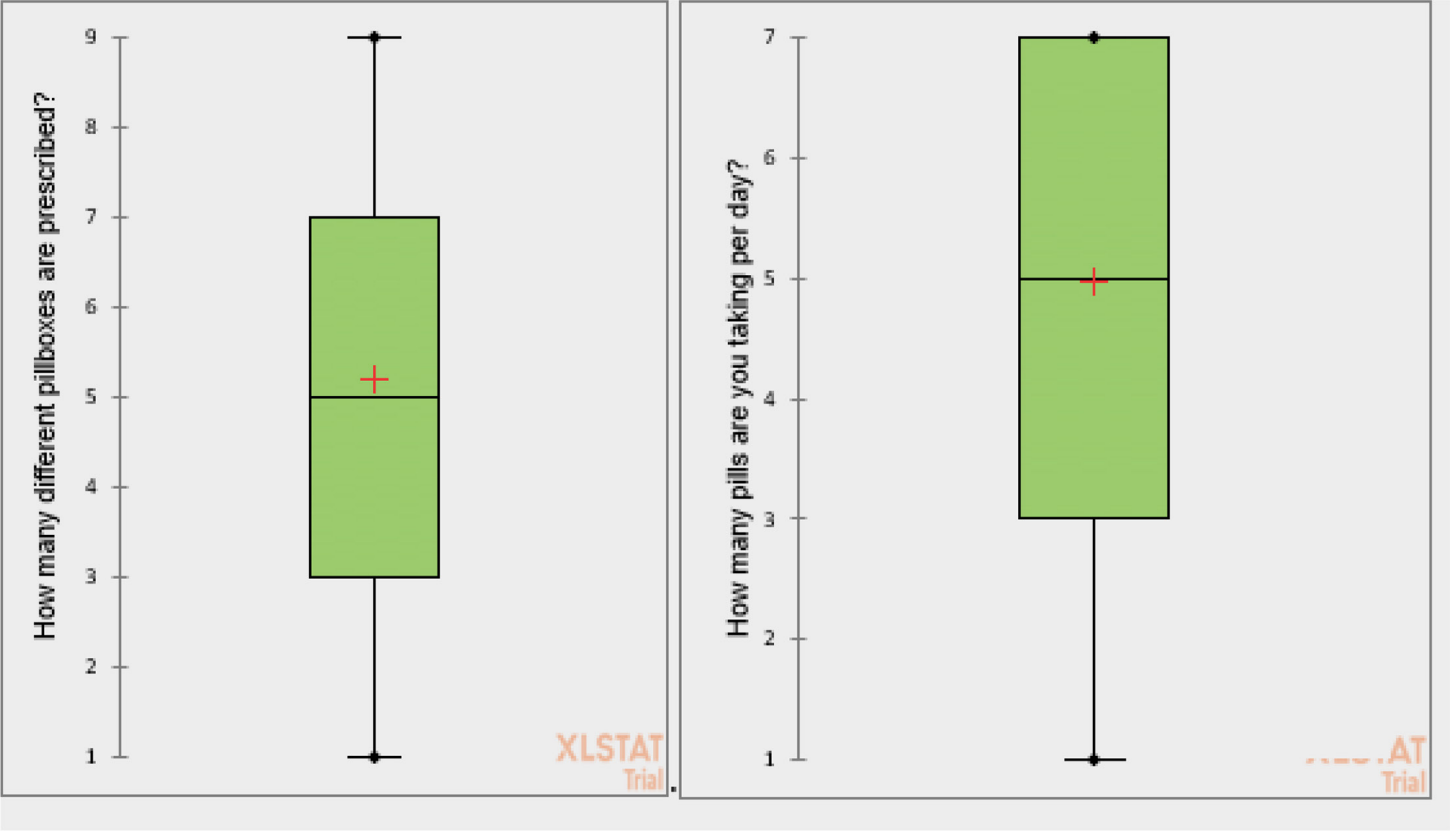
Number of pillboxes prescribed (lines of prescription) and pills taken per day

**Figure 3.**
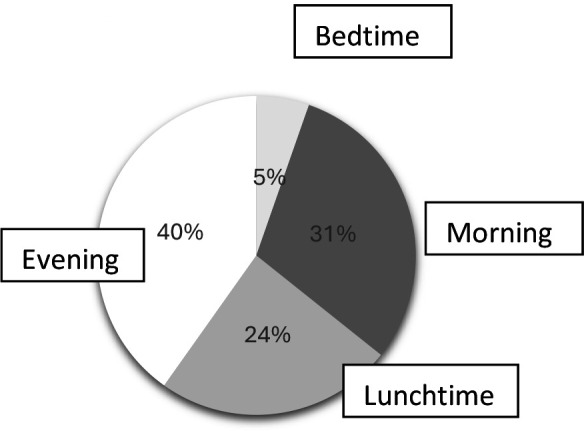
Frequency and moments of omissions throughout a day

The number of boxes prescribed, the number of medicines taken per day, and the moments of omissions are shown in Figure 2 and Figure 3. Neither occupation (*P* = 0.12) nor use of a pillbox (*P* = 0.811) influenced the frequency of omissions.

Regarding knowledge of the medicines listed in their prescription, 79% said they knew all of them, 2% none of them, and 19% some of them. Knowledge of treatment was independent of profession (*P* = 0.5).

Forty-three per cent used a pillbox and 95% of them were satisfied and felt it met their needs. There was a link between pillbox use and sex (*P* = 0.03), with female patients tending to use pillboxes less often than male patients. There was a link between the number of times medication was taken and the use of a pillbox (χ^2^ test with a *P* value of <0.0001).

Fifty-eight percent reported missing pills without a pillbox. Among those who dit not use a pillbox, 42% decled never forget their medication, compared to 35% who used a pillbox.

Treatments were prepared in advance for 51% of responders, including 64% on a weekly basis, 30% daily, and 5% monthly. When medicines were prepared on a weekly basis, Sunday and Monday were the most common days chosen (31%).

## Discussion

### Summary

In this quantitative study, which focused on the primary care setting, we assessed how 180 patients with CDs managed their medicines at home. We confirmed that unintentional omissions are common among people with chronic illness. These can be identified and reduced by suggesting reminder tools (pillbox, telephone alarm, application) adapted to their needs and preferences. In our sample, the frequency of omissions remained the same with or without a pillbox, which is important for the GP during the long-term management of people with chronic illness.

### Strengths and limitations

Our study sample is representative of the French population, living with one or more chronic condition.^5^

The inclusion criteria were simple, and the populations studied represented people consulting general practices, which facilitated recruitment and resulted in a response rate of 60%, which is remarkable for a survey among both French GPs and their patients, who are regularly invited to participate in surveys, and may have not much time to participate in surveys. The anonymous questionnaire could be completed in the waiting room, so people could answer freely and without the impression of direct judgement by the doctor or with the doctor’s help, which made it possible to open a dialogue about compliance. It was interesting to ask doctors if they had themselves a chronic condition and if they used a pillbox. There may have been reporting and recall bias, as well as fatigue in filling in the questionnaire, which was relatively long. A selection bias for the GPs choosing their patients and the limitations of questionnaire surveys without other checks on actual behaviour as well as declaration bias are possible. Furthermore, patients who needed assistance from the GP to complete the survey may have selected answers according to social desirability.

### Comparison with existing literature

According to the National Institute of Statistics and Economic Studies (INSEE), the population with a CD in France represents about 41% of women and 39% of men, giving an odd ratio of 0.95%.^5^ With an odd ratio of 0.91, our sample population is comparable with the general population.

The median age was 66.5 years, with the majority aged >65 years (63%), 47% aged between 55 years and 75 years, and 38% aged >75 years. Our results are in line with those of INSEE in 2017, with almost half of patients aged 55–59 years and more than 70% of men and women aged ≥75 years.^5^

For the 55–75-year age group, we found a good level of representativeness, unlike the ≥75-year age group. This is owing to recruitment bias, as older patients are more dependent and more likely to attend the practice.

Our sample included a higher proportion of retired people than in the general population, probably owing to selection bias within the practice, where they consult more frequently than people who are still working.

Regarding the place of storage and its risks, a qualitative study in New Zealand showed that the kitchen and bathroom were the most reported locations, ahead of convenience and the desire not to forget, despite the high temperatures and humidity that make these rooms unsuitable for storage.^11^ In our study, we confirm these locations.

Temperature variations, humidity problems, and lack of airtightness lead to deterioration in the chemical composition of medicines. Inappropriate storage locations and methods were also identified in the study by Beuscart *et al*. They surveyed 1370 patients with an average age of 81.5 years who took an average of 9.3 medications daily. Of these, 743 (54.2%) had a home visit with examination of the home medicine cabinet. Poorly located cupboards (in 15% of inspections), medication storage problems (21.7%), expired medications (40.7%), potentially inappropriate medications (15.0%), several different generic versions of the same medication (19.9%), and redundant medications (20.4%) were identified.^12^

The multiplication of storage locations, as reported by nine among our participants, can lead to forgetfulness and the risk of errors.

Regarding intentional and unintentional non-adherence, non-adherence is a common phenomenon with potentially serious medical and economic consequences, and can be unintentional or intentional. A person may deliberately choose not to take their treatment (intentional non-adherence).^13^ Fear of side effects, not feeling the need to take their medication, or just not wanting to take it can be reasons for this phenomenon.

In our study, 61% of participants reported forgetfulness or omission, the majority of which appeared to be unintentional, based on the open-ended responses (Table 3).

A study of 134 patients with type 2 diabetes showed that the strength of the medication-taking habit was significantly associated with medication adherence.^13^

An online survey of 3000 people with cardiovascular disease showed that habitual compliance (also known as unintentional compliance) was associated with trust in the doctor and having enough information about the disease and treatment.^13^ In our study, 97% of patients took their treatment routinely "out of habits". Few validated methods exist for measuring adherence directly at home.^14^

Regarding the pillbox and other tools, many tools exist and are being developed to help patients promote their autonomy and manage their treatment of their CD. The patient-centred or patient empowerment approach encourages us to observe and listen to patients' needs and to propose appropriate solutions.^15^

In the present study, 58% of the population surveyed reported missing pills without a pillbox. Of those who did not use a pillbox, 42% never forgot to take their medication, compared with 35% of those who used a pillbox. This result is surprising and contradicts the expectation that pillbox users would be more compliant than non-users.^16^

The pillbox is a management tool. They come in different shapes and sizes, depending on the patient’s needs and preferences; for example, the daily pillbox usually contains four compartments for taking morning, noon, and evening medication; the weekly pillbox usually contains seven compartments with four compartments; the connected pillbox allows you to plan your treatment and check that you are taking your medication as prescribed, and if you forget to take your medication, you can alert the patient or someone close to you by sound reminder, text message, email or notification on your tablet. Some electronic pillboxes are linked to a service that alerts the pharmacist if the pillbox is not opened.^17^ A pilot study has been conducted on the acceptability of using an intelligent pillbox to improve patient compliance in primary care.^18^

There are many other management tools available, such as an Excel spreadsheet or simply using free paper. Several telephone applications for reminding people to take their treatment exist and combine different services, such as reminders, automatic integration with patients' electronic diaries, and recording of information about the patient and the context in which the treatment is being taken.

Mobile health (mHealth) is now the subject of a best-practice reference framework on applications and connected objects in health, published by the French National Authority fo Health (Haute Autorité de Santé).^19^

### Implications for research and practice

Among our participants, 15% declared conflicts and 30% burden owing to MM at home. This is an important information for the GP, who must consider these psycho-social aspects of MM, because they can reduce compliance.

In our sample, 61% of responders forgot to take their medication rarely (38%), sometimes (21%), or often (2%) and 19% only knew some of the medication on their prescription.

It is important to review and to optimise the practicalities of prescribing together with the patients through a person-centred and shared decision-making process.

When prescribing and renewing prescriptions, GPs should remind patients of the therapeutic purpose of each medicine. Prescriptions must be written correctly and legible as patients rely on them to prepare their pillboxes and sometimes as their only aid to compliance. As a result, poorly written or illegible prescriptions can be a source of error.

Some business software packages, are configured to print a pillbox in tabular form, which can be given to the patient along with the prescription. More and more networked tools are being developed.

Ideally, the number of boxes prescribed should be reduced to the minimum necessary, as in the case of treatments that combine two active molecules, particularly for high blood pressure. Wherever possible, taking medication in the morning or at lunch seems to reduce the number of missed doses, which are mostly forgotten in the evening (40%). Offering pillboxes suitable for travelling, or miniature pillboxes for outings, would reduce the number of forgotten doses during short or long trips (19%).

Finally, whenever possible, it is useful and appropriate to organise a home visit to find out how to store and prepare medicines, how to use reminders, and how to take them orally (alone or with assistance), as certain conditions, such as rheumatoid arthritis or Parkinson’s disease, can make it technically difficult to take a medicine (difficult to hold).

In France, home visits by GPs have become increasingly rare since 2005. However, the benefits of preventive home visits for the older population with chronic illness have been demonstrated by studies in primary care settings. Frese *et al* showed a reduction in mortality and a 22% lower risk of admission to a nursing home.^20^ Limatta *et al* highlighted positive effects on health-related quality of life at no additional cost from multiprofessional preventive home visits.^21^

The GIRERD questionnaire can be used during regular follow-up visits to assess adherence using six simple questions.^22^ In addition, difficulties with MM at home (from reading the prescription to storing, preparing the pillbox, and taking the medication) can be easily identified by the clinical pharmacist during the pharmacy visit, thus promoting medication adherence in CD.^12,23^

In conclusion, unintentional forgetfulness is common among people with chronic illness and can be easily identified and corrected by discussing home MM during consultation. If necessary, offering tools (pillbox, telephone alarm, application) adapted to the personal needs and preferences of people taking a daily oral treatment, can contribute to a better compliance.

As far as the frequency of omissions remains the same with or without a pillbox, repeated intervention by the GP is essential to optimise adherence and to remind the indication for each medication. Finally, the GPs must be aware (and explore) that MM at home can be a source of possible burden for patients themselves and/or their relatives as well as for conflicts, which are likely to reduce compliance.

## Data

The dataset relied on in this article is available from the corresponding author on reasonable request.
